# Protein phosphorylation networks in *Baylisascaris procyonis* revealed by phosphoproteomic analysis

**DOI:** 10.1186/s13071-025-06949-y

**Published:** 2025-07-28

**Authors:** Qin Meng, Zhikang Li, Qiguan Qiu, Shuyu Chen, Haiyan Gong, Xiaoruo Tan, Xiaoheng Liu, Zhaoguo Chen, Wei Liu

**Affiliations:** 1 Research Center for Parasites & Vectors, College of Veterinary Medicine, Hunan Agricultural University, Changsha, 410128 Hunan People’s Republic of China; 2 Changsha Ecological Zoo, Changsha, Hunan 410119 People’s Republic of China; 3 Animal Husbandry and Fisheries Affairs Center of Huaihua, Huaihua, 418000 China; 4 Key Laboratory of Animal Parasitology of Ministry of Agriculture, Laboratory of Quality and Safety Risk Assessment for Animal Products On Biohazards (Shanghai) of Ministry of Agriculture, Shanghai Veterinary Research Institute, Chinese Academy of Agricultural Sciences, Shanghai, 200241 People’s Republic of China

**Keywords:** *Baylisascaris procyonis*, Raccoon, Liquid chromatography-mass spectrometry, Phosphoproteome, Function, Signaling pathway

## Abstract

**Background:**

*Baylisascaris procyonis* is an intestinal ascarid worm that parasitizes in raccoons and causes fatal neural, visceral, and ocular larva migrans in humans. Phosphorylated proteins and protein kinases have been studied as vaccine and drug target candidates against parasitic infections. However, no data are available on protein phosphorylation in the raccoon roundworm.

**Methods:**

In this study, the entire proteome of adult *B. procyonis* was enzymatically digested. Then, phosphopeptides were enriched using immobilized metal affinity chromatography (IMAC) and analyzed by liquid chromatography-mass spectrometry (LC-MS/MS).

**Results:**

Our phosphoproteome analysis displayed 854 unique phosphorylation sites mapped to 450 proteins in *B. procyonis* (3308 phosphopeptides total). The annotated phosphoproteins were associated with various biological processes, including cytoskeletal remodeling, supramolecular complex assembly, and developmental regulation. The phosphopeptide functional enrichment revealed that *B. procyonis* phosphoproteins were mostly involved in the cytoskeleton cellular compartment, protein binding molecular function, and multiple biological processes, including regulating supramolecular fiber and cytoskeleton organization and assembling cellular protein-containing complexes and organelles. The significantly enriched pathways of phosphoproteins included the insulin signaling pathway, tight junction, endocytosis, longevity-regulating, glycolysis/gluconeogenesis, and apelin signaling pathways. Domain analysis revealed that the Src homology 3 domain was significantly enriched.

**Conclusions:**

This study presents the first phosphoproteomic landscape of *B. procyonis*, elucidating phosphorylation-mediated regulation of cytoskeletal dynamics, host interaction pathways, and metabolic adaptations. The identified 450 phosphoproteins and enriched functional domains establish a foundation for targeting conserved mechanisms critical to *B. procyonis* survival.

**Graphical Abstract:**

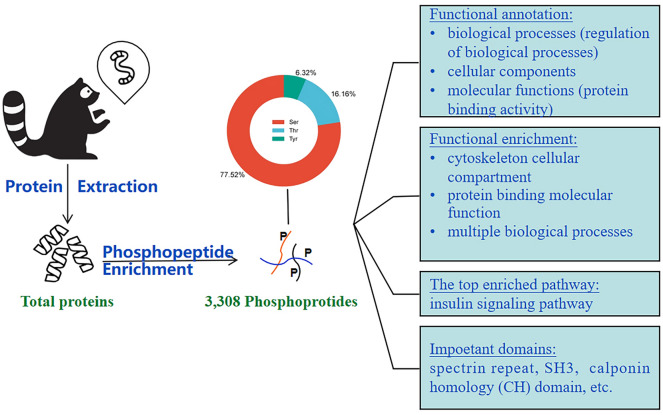

**Supplementary Information:**

The online version contains supplementary material available at 10.1186/s13071-025-06949-y.

## Background

*Baylisascaris* spp. are zoonotic intestinal roundworms belonging to the family Ascarididae with a wide distribution in Europe and Asia. The parasites in this genus can infect about 150 kinds of animals, including raccoons, skunks, bears, badgers, and even humans [1]; among these, the infection rate in raccoons is very high (e.g. 68–82% in the Midwestern USA) [1]. Young raccoons are apt to acquire the infection through ingestion of infective eggs in feces, while the adults are typically infected through consumption of larvae-containing paratenic hosts, such as rodents [2–4]. With the defecation of the infected animals, 20,000–26,000 eggs per gram of feces are released into the environment [2], which can survive and remain infective for several years [5], thus increasing the risk of *Baylisascaris procyonis* zoonotic transmission by environmental contamination [6–8]. After ingestion of infective eggs by humans, the developed larvae can result in fatal neural, visceral, and ocular larva migrans [9, 10]. Compared to adults, young children aged 1–4 years are more vulnerable to intense infection because of poor hygiene and pica. On the other hand, raccoons frequently migrate from rural to urban areas and wander around human-dominated settings, also increasing the risk of infection in city residents. To date, most cases have been reported in North America (Canada and the US) because of the wide spread of raccoons [11–13]. More seriously, the treatment in most cases only leads to poor alleviation of the *Baylisascaris*-caused neurological symptoms, which can be fatal, necessitating the development of new drug or vaccine strategies to control and prevent this parasite.

Protein post-translational modifications (PTMs) are the fast regulatory processes and biochemical changes that occur during or after protein translation [14]. Reversible protein phosphorylation at serine, threonine, and tyrosine residues is one of the essential PTMs regulating several cellular processes and signal pathways [15]. The protein phosphorylation and dephosphorylation processes are activated by protein kinases and phosphatases, which are responsible for phosphoryl group addition or removal from substrates [16, 17]. Some protein kinases have been characterized as possible drug targets for zoonotic parasites, such as *Schistosoma*, *Toxoplasma*, and *Leishmania* [18–20]. Recently, phosphoproteome studies in several organisms, including parasites, have been carried out to identify thousands of unique protein phosphorylation sites using phosphopeptide enrichment coupled with mass spectrometry. This provided a new insight into the characteristics and function of the phosphoproteins [21, 22]. Goyal et al. revealed that *Plasmodium falciparum* canonical histone H2A is phosphorylated when exposed to DNA damage sources [23]. Zhang et al. found that *Plasmodium* and *Toxoplasma α* subunit of eukaryotic translation initiation factor 2 (eIF2α) kinases and phosphatases were essential for sufficient development and adaptation to cellular stresses throughout the life cycle. Thus, inhibitors of these kinases may be potential targets to develop novel malaria and toxoplasmosis therapies [24]. Comparison of the phosphoproteomes of *Leishmania donovani* promastigotes to amastigotes suggested that most of the phosphorylated sites were detected at specific life cycle stages and might participate in the regulation of the process [25]. Phosphoproteomic analyses of male and female *Schistosoma mekongi* suggested that protein phosphorylation was crucial for the growth, development, and reproduction of the parasite [26]. A phosphoproteomic analysis of *Spirometra erinaceieuropaei* spargana revealed 3228 phosphopeptides, mainly involved in the signaling molecular function, the cytoskeleton cellular compartment, and several biological processes [21]. However, there is still no report on protein phosphorylation in *B. procyonis*.

In this study, the total released phosphopeptides were investigated using immobilized metal affinity chromatography (IMAC) combined with liquid chromatography-mass spectrometry (LC-MS/MS) to explore the phosphorylation processes in *B. procyonis*, and 3277 phosphorylation sites (p-sites) were detected in the 1059 isolated proteins. These results might contribute to a deep understanding of the functions of protein phosphorylation in *B. procyonis*.

## Methods

### Sample isolation

Adults of *B. procyonis* were isolated from the intestine of a raccoon from Changsha, Hunan Province, China, using the method of Camp et al. [27], and then thoroughly rinsed in pre-chilled phosphate-buffered saline (pH 7.4). Species identification was confirmed by morphological examination and molecular analysis (Additional file 2: Text S1, Fig. S2). Three biological replicates (*n* = 10 each) were performed to improve the reliability of identifying phosphorylation sites, including two replicates with the soluble protein fraction and one with the insoluble protein fraction. All samples were placed in liquid nitrogen for 1 h before storage at − 80 °C for future use.

### Protein extraction

The frozen specimens were mixed with liquid nitrogen in a pre-cooled mortar, fully ground into powder, and transferred to a new 5-ml centrifuge tube. Each sample was homogenized with four times the volume of lysis buffer, consisting of 10 mM dithiothreitol (Sigma-Aldrich, MO, USA) and 1% protease inhibitor (Abcam, Cambridge, UK) and 1% phosphatase inhibitor (Millipore, Massachusetts, USA), before ultra-sonication. Then, the lysates were centrifuged (4 °C, 5500 × g, 10 min) to eliminate the cell debris. The supernatants were collected, and five times the volume of 0.1 M acetone/methanol was added for overnight precipitation. The precipitate was washed sequentially with methanol and acetone. Finally, the precipitate was resuspended in 8 M urea, and the protein concentration was measured with a BCA assay kit.

### Protein digestion

For enzymatic digestion, 1000 μg of protein per sample was taken, and the volume was adjusted to be consistent using lysis buffer. Trichloroacetic acid (TCA, Sigma-Aldrich, St Louis, MO, USA) was slowly added to a final concentration of 20% (w/v), and the mixture was vortexed to ensure homogeneity. The solution was incubated at 4 °C for 2 h to allow protein precipitation. The samples were centrifuged at 4500 × g for 5 min, and the supernatant was discarded. The pellet was washed 2–3 times with pre-chilled acetone (Zhejiang Hannuo Chemical Technology Company, Zhejiang, China) and air-dried. After drying, the pellet was resuspended in 200 mM triethylammonium bicarbonate (TEAB, Sigma-Aldrich) buffer, and the solution was sonicated to thoroughly dissolve the pellet. Trypsin (Promega, Madison, WI, USA) was added at a 1:50 mass ratio (enzyme: protein, m/m), and the mixture was digested overnight at 37 °C.

### Phosphopeptide enrichment

The dried peptides were dissolved in 50% acetonitrile (Thermo Fisher Scientific, Waltham, MA, USA)/0.5% acetic acid (Hangzhou Gaojing Fine Chemical, Zhejiang, China) loading buffer and then incubated with pre-washed IMAC microspheres (Thermo Fisher Scientific) with shaking. After incubation, IMAC microspheres were rinsed three times in 50% acetonitrile/0.5% acetic acid and then in 30% acetonitrile/0.1% trifluoroacetic acid (Sigma-Aldrich, MO, USA) to eliminate the non-specifically adsorbed peptides. The enriched phosphopeptides were then eluted using a 10% NH_4_OH-containing elution buffer with shaking. After collecting and lyophilizing the eluates, the enriched phosphopeptides were desalted according to the instructions of C18 ZipTips (Merck, Darmstadt, Germany) and then lyophilized for liquid chromatography-tandem mass spectrometry (LC-MS/MS) analysis.

### LC-MS/MS analysis

LC-MS/MS analysis was performed according to established procedures described in published protocols [28, 29]. Enriched phosphopeptides were dissolved in mobile phase A (0.1% formic acid and 2% acetonitrile) and quantified using the Pierce^™^ Quantitative Colorimetric Peptide Assay (Thermo Scientific, Waltham, MA, USA). Based on this quantification, 0.5 μg peptides per injection were loaded onto a reversed-phase analytical column (25 cm × 75 μm; Thermo Fisher Scientific). Each sample was injected once without technical replicates. Peptides were separated on an EASY-nLC 1200 UPLC system (Thermo Fisher Scientific) using a mobile phase B (0.1% formic acid in 100% acetonitrile) with the following gradient: from 0 to 74 min, 2–22% B; from 74 to 80 min, 22–35% B; from 80 to 85 min, 35–80% B. They were maintained at 80% B from 85 to 90 min at a constant flow rate of 450 nl/min.

Separated peptides were analyzed on a timsTOF Pro mass spectrometer (Bruker Daltonics, Billerica, MA, USA) with a nano-electrospray ion source (1.6 kV). Precursor ions (charge states 0–5) were detected in high-resolution TOF mode (m/z 100–1700). MS/MS used PASEF mode (10 spectra/MS cycle) with 30-s dynamic exclusion.

### Database search

MaxQuant v1.6.15.0 software was used to retrieve the MS/MS spectra. The search parameters were set as follows: the database comprised a custom Ascarididae-specific protein library constructed from the *B. procyonis* genome (Additional file 3: Dataset S1, containing 18,499 sequences), supplemented with a decoy database to estimate the false discovery rate (FDR) caused by random matches. Additionally, common contaminant proteins were included to minimize the impact of contaminant proteins on the identification results. Trypsin/P was selected as the enzyme digestion mode, with two missing cut points. The peptide segment minimum length was seven residues, whereas the maximum modification number was five residues. The first search was set to 20 ppm, while the main search was set to 5 ppm, with a 0.02-Da mass error tolerance for secondary fragment ions. Cysteine alkylation was designed as a fixed modification reference. Methionine oxidation, protein N-terminal acetylation, and serine, threonine, and tyrosine phosphorylation were combined as alterable modification references. The protein and peptide-spectrum match (PSM) identification had a 1% FDR.

### Motif analysis

The motif-x online tool (http://motif-x.med.harvard.edu/motif-x.html) was used to determine the composition of the amino acid residues flanking the phosphosites. This tool tested protein sequence models of amino acids at phospho-21-mers-specific positions (10 residues upstream and downstream phosphosites) [30]. The protein sequence database was employed as background parameter, with the other parameters set to the default settings. A specific sequence was considered a motif of the modified peptide when the number of peptides in this sequence exceeded 20, with a statistical *P*-value < 0.000001.

### Phosphoproteome functional annotation

BLAST analyses were conducted using the Blast2GO platform [31]. Phosphoprotein functional enrichment analyses were conducted using the BiNGO plug-in [32] in the Cytoscape platform [33]. The significantly enriched GO terms in the phosphoproteome were compared to the “both non-phosphoproteins and phosphoproteins” group. The hypergeometric distribution of phosphoproteins in a certain branch(s) in the GO classification was determined depending on the selected phosphoproteins. The GO analyses provided a hypothetical *P*-value for each GO term. A low *P*-value indicates the enrichment of the GO differential genes. The enrichment of the differentially modified proteins was tested against the entire set of determined proteins using Fisher’s exact test. The InterPro database (http://www.ebi.ac.uk/interpro/) was searched for each phosphoprotein category to predict the presence of domains, and Fisher’s exact test was applied. For the protein domains, a corrected *P*-value of < 0.05 was regarded as significant. The most closely referenced taxonomy was [7209: Loa loa], with detailed information accessible through the following URL: https://www.uniprot.org/taxonomy/7209.

### Phosphoprotein pathway analysis

The enriched pathways were identified using the Kyoto Encyclopedia of Genes and Genomes (KEGG) database (https://www.genome.jp/kegg/). Depending on the derived data from this database, the classified pathways were displayed in hierarchical categories. The most closely referenced taxonomy was [7209: Loa loa], with detailed information accessible through the following URL: https://www.uniprot.org/taxonomy/7209.

### Enrichment-based clustering

The hierarchical clustering analyses were conducted depending on differentially expressed protein functional classifications, such as GO, domains, and pathways. All the recovered categories were first compiled after enrichment with their *P*-values. Then, the categories enriched in one or more clusters, with a *P*-value < 0.05, were filtered. The filtered *P*-value matrices were converted into the function *x* = −log10 (*P*-value). The *x* values of each functional category were transformed into *z*-scores. Then, the *z*-scores were gathered using Genesis one-way hierarchical clustering (Euclidean distance and average linkage clustering). Finally, the cluster membership was visualized by drawing a heat map with the “heatmap 2” function of the “gplots” R package.

### Phosphoprotein interaction network analysis

All *B. procyonis* protein-protein interaction data that matched *B. procyonis* phosphoproteins identified in this study were downloaded from the STRING database v.11. The most closely referenced organisms were *Toxocara canis*. Then, all these data were employed to construct the phosphoproteome interaction network with a 0.7 minimum confidence level, and we visualized this network in the R package “networkD3”.caen.

## Results and discussion

### Establishment of *B. procyonis* phosphoproteome

Phosphoproteome analysis facilitated the understanding of the function of complex cell signaling networks and phosphorylation in various organisms [22]. Protein phosphorylation or dephosphorylation, monitored by protein phosphatases and kinases, contributed to different cellular processes, maintaining parasite cell homeostasis [16]. In the present study, the phosphoproteome of the zoonotic parasite *B. procyonis* was investigated for the first time to our knowledge. As a result, a total of 3308 phosphopeptides (Additional file 4: Table S1) and 854 phosphorylation sites were identified in 450 isolated proteins. We deposited MS data in the ProteomeXchange under the identifier PXD066529 (http://proteomecentral.proteomexchange.org/ dataset/PXD001684). Additional file 5: Table S2 shows the identification algorithm scores and PTM scores. The number of phosphopeptides detected in *B. procyonis* was similar to that in other nematodes, such as *Haemonchus contortus* (4406 phosphopeptides in 1804 proteins) [17], *Caenorhabditis elegans* (6780 phosphopeptides in 2373 proteins) [34], and *Pristionchus pacificus* (6809 phosphopeptides in 2508 proteins) [35]. Among the identified phosphoproteins, glutathione S-transferase (GST) was considered a promising vaccine candidate against nematodes [36]. In eukaryotes, glycogen synthase kinase 3 (GSK3) phosphorylates glycogen synthase and serves as a downstream element in the Wnt pathway during embryogenesis in mosquitoes [37], and it participates in the regulation of embryogenesis and oogenesis in *Rhodnius prolixus* [38]. In *Schistosoma japonicum,* GSK3 was overexpressed in eggs [39]. The GSK3*β* RNAi significantly reduced the kinase activity and cell life span of the parasite, indicating that GSK3*β*-dependent phosphorylation was vital for the worm’s survival [39]. However, the role of GSK3 phosphorylation in *B. procyonis* development and survival needs to be further explored. The accession had chaperone activity and contributed to the development, stress response, and immune response in helminth and protozoan parasites [40].

**Fig. 1:**
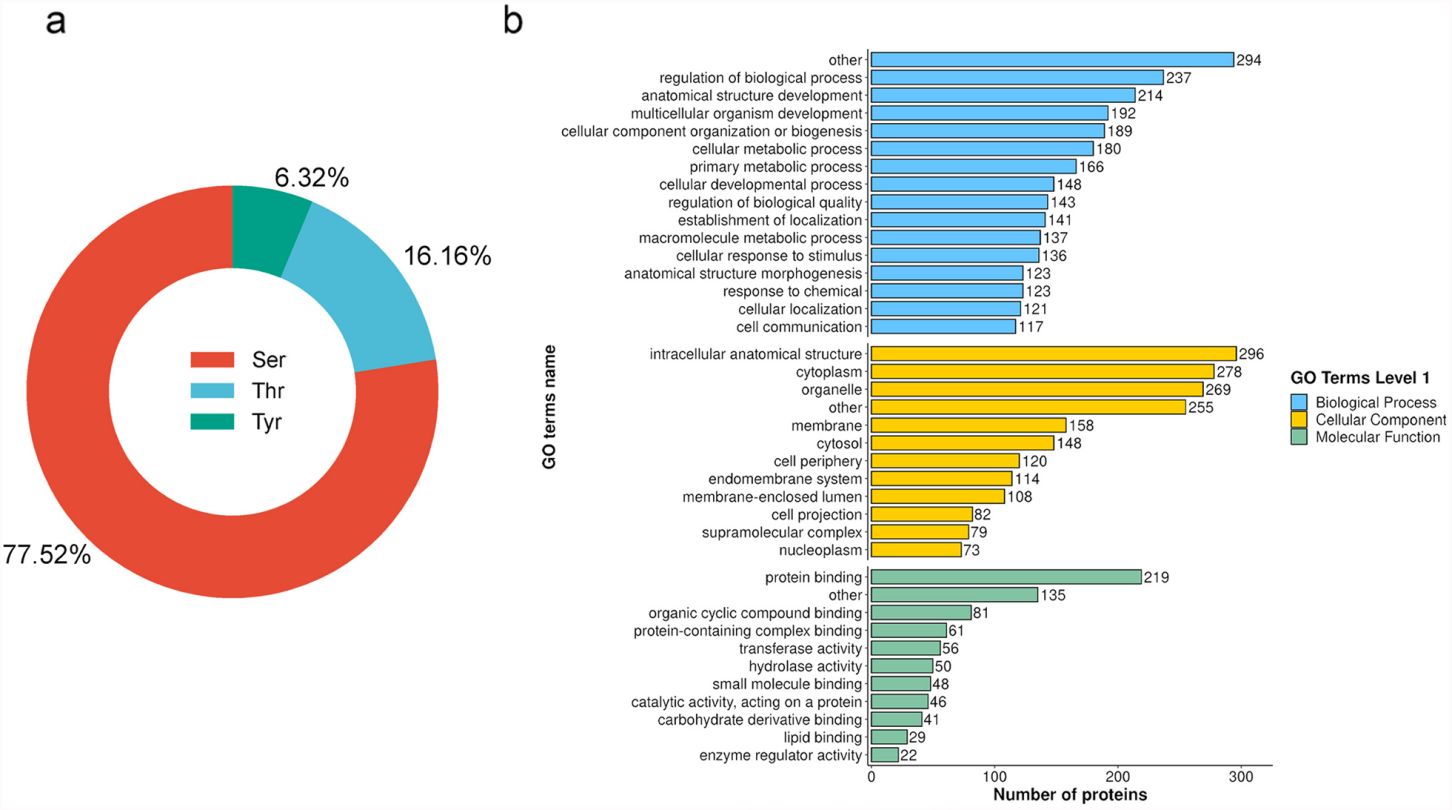
General description of *B. procyonis* phosphoproteome data. (**a**) Pie chart illustrating the distribution of phosphorylation sites identified. (**b**) GO term analysis of *B. procyonis* phosphoproteins. Blast2GO was used for GO annotation and categorization

In *B. procyonis*, phosphoserines showed the highest proportion (77.52%) in the 854 phosphorylation sites, followed by phosphothreonines (16.16%) and phosphotyrosines (6.32%) (Fig. 1a). Previous studies revealed a similar phosphoserine/phosphothreonine/phosphotyrosine ratio in *Plasmodium falciparum* (84.4/13.2/2.4%), *Trypanosoma brucei* (75/21.5/3.5%), *Haemonchus contortus* (85/14/1%) [17], *Fasciola gigantica* (87.3/11.7/1.0%) [16], *P. pacificus* (87.8/11.1/1.06%) [35], *C. elegans* (80/18.2/1.8%) [34], and *Spirometra erinaceieuropaei* (87/10/3%) [21, 41–43]. However, a higher percentage of phosphotyrosine (12%) was detected in *Schistosoma mansoni* [22]. The phosphorylation on tyrosine was mostly associated with a strong negative and transient regulation on the tyrosine kinases, in vivo [44], with a rare structural modification on the protein. In the absence of technical bias toward p-tyrosine capture, the high abundance of *S. mansoni* phosphotyrosines might be explained by the higher activity and more intense regulatory action of the tyrosine kinase in *S. mansoni* compared to other eukaryotes [22].

Functional annotation conducted via Blast2GO revealed that differentially expressed proteins were predominantly enriched in three categories: biological processes, cellular components, and molecular functions. In the biological processes category, the annotated phosphoproteins contributed to various biological processes, including regulation of biological processes, anatomical structure development, multicellular organism development, cellular component organization or biogenesis, cellular metabolic process, primary metabolic processes, cellular developmental processes, regulation of biological quality, establishment of localization, macromolecule metabolic processes, cellular response to stimulus, anatomical structure morphogenesis, response to chemical and cellular localization, and cell communication (Fig. 1b). This finding may be explained by the pleiotropic nature of phosphorylated proteins. In the molecular function GO category, the highest percentage of phosphoproteins was involved in the protein binding activity, followed by other organic cyclic compound binding, protein-containing complex binding, transferase activity, hydrolase activity, small molecule binding, catalytic activity, acting on a protein, carbohydrate derivative binding, lipid binding, and enzyme regulator activity (Additional file 6: Table S3). This was consistent with observations of several other parasites, such as *F. gigantica* [16], *H. contortus* [17], and *Schistosoma mansoni* [22], etc.

According to the prediction of the phosphoprotein subcellular localization using the Wolfpsort software (Additional file 7: Table S4), almost 1/3 of the phosphorylated protein was assumed in the nucleus (34.44%), followed by the cytoplasm (31.11%), plasma membrane (10.67%), mitochondria (8.89%), and extracellular matrix (6%), which indicated a similar trend to a previous study by detecting a small proportion of phosphoproteins in the extracellular side. This result also supported the reliability of our analysis because extracellular molecules are not frequently regulated by phosphorylation processes [16].

The establishment of the *B. procyonis* phosphoproteome lays a foundation for probing phosphorylation-driven adaptations in this zoonotic parasite. To advance these findings, future studies could prioritize temporal resolution by mapping phosphorylation dynamics across developmental stages or under host-derived stressors.

### Analysis of amino acid residues flanking the phosphorylation sites

Amino acids in phospho-21-mers in *B. procyonis* phosphoproteins were searched to clarify the characteristics of amino acid residues surrounding the p-sites. After determining p-sites using the motif-x tool, we categorized five motifs, composed of four phosphoserine and one phosphothreonine motifs (Additional file 8: Table S5). The absence of detectable phosphotyrosine motifs could be related to the low number of phosphotyrosine sites in *B. procyonis* [21]. Among phosphoserine motifs, the "…SP…" motif exhibited the highest kinase specificity with a 3.3-fold increase. The predominant phosphothreonine motif "…TP…" showed the strongest enrichment (4.8-fold increase). These motifs included in the basic protein kinases, such as “…R…S…” and “…R…TP…” motifs, represent signatures for Ca^2+^/calmodulin-dependent protein kinases (CAMK). CAMK4 of *P. falciparum* was involved in regulating parasite invasion [45]. Calmodulins (CaMs) were a critical molecule for protein kinase phosphorylation, gene expression, and calcium transport [46]. CaMs also participated in the egg hatching and larval development of *S. mansoni* [47], juvenile fluke growth and motility of *F. hepatica* [48], and neuronal activity of *C. elegans* [49]. “…SP…” is a motif for proline-directed protein kinases for mitogen-activated protein kinase (MAPK/ERK) cells. ERK was involved in adult mating, egg laying, movement [50], and host invasion of *S. mansoni* [51] as well as stress regulation in *Leishmania* [52]. Further studies should elucidate the functional roles of these kinase systems in *B. procyonis* biology.

### Functional enrichment analysis of phosphoproteins

**Fig. 2:**
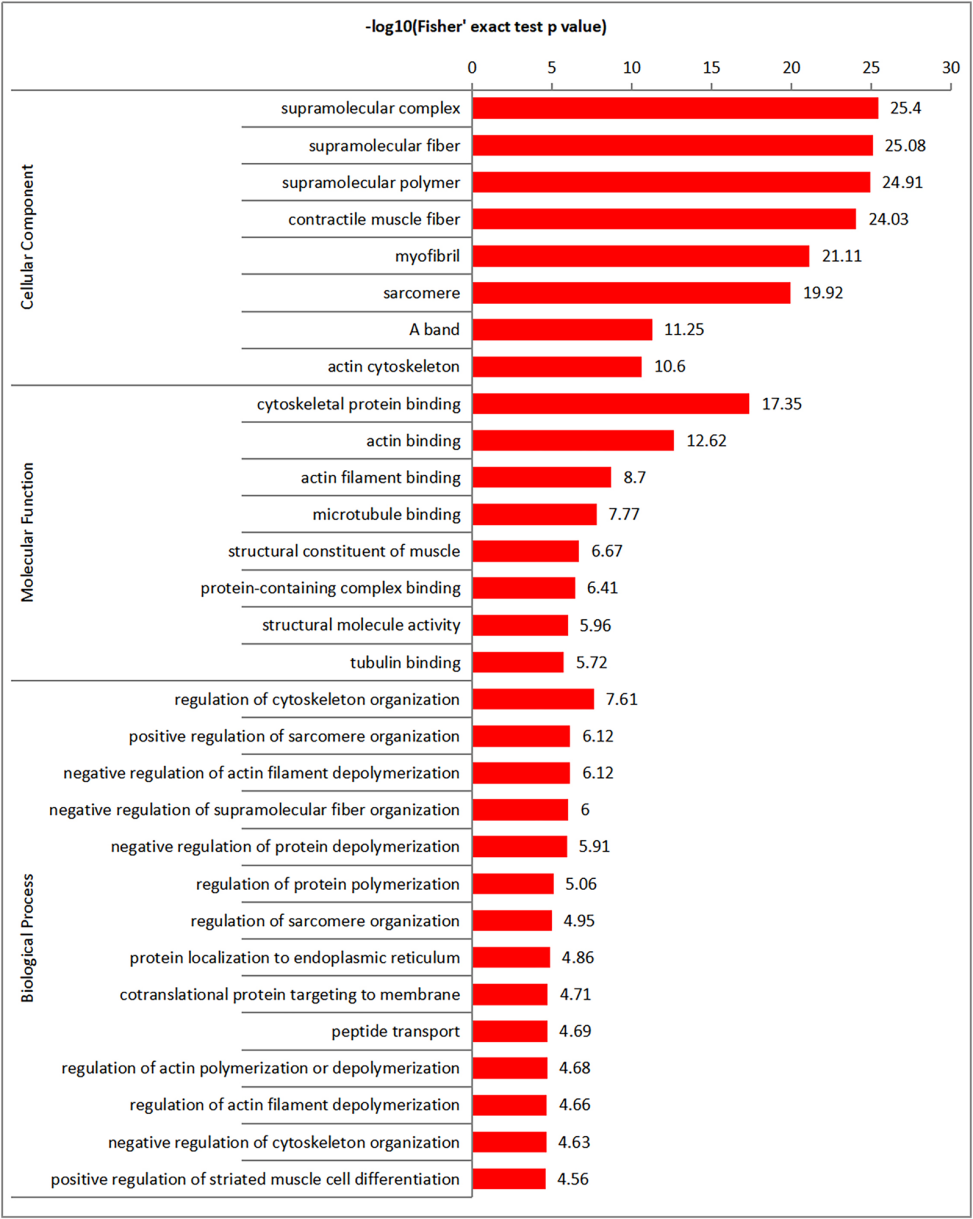
GO enrichment analysis of *Baylisascaris procyonis* phosphoproteins. The GO categories were biological process, cellular components, and molecular functions

The functional enrichment analysis of phosphoproteins reveals a highly coordinated signaling network predominantly targeting the architecture and dynamics of supramolecular structures, particularly within the cytoskeleton and contractile apparatus. As shown in Fig. 2, the top six entries (−log10(*P*) > 19.9) all belong to cellular components (CC) and are highly concentrated in large supramolecular complexes, fibrous structures, and muscle contraction units: supramolecular complex (25.4), supramolecular fiber (25.08), supramolecular polymer (24.91), contractile muscle fiber (24.03), myofibril (21.11), and sarcomere (19.92) (Additional file 9: Table S6).

Molecular functions (MFs) are predominantly focused on cytoskeleton-related binding. The most significantly enriched molecular functions are cytoskeletal protein binding (17.35), actin binding (12.62), actin filament binding (8.7), and microtubule binding (7.77). The most significantly regulated biological processes (BP) primarily revolve around the organization and dynamics of the cytoskeleton and sarcomere: regulation of cytoskeleton organization (7.61), positive regulation of sarcomere organization (6.12), negative regulation of actin filament depolymerization (6.12), negative regulation of supramolecular fiber organization (6.00), and negative regulation of protein depolymerization (5.91).

Collectively, these results demonstrate that phosphorylation acts as a master regulatory switch, targeting structural constituents and binding proteins within supramolecular complexes, fibers, and muscle units to precisely control their stability, assembly kinetics, and functional dynamics, crucial for cytoskeletal remodeling and muscle function. The enrichment of positive regulation of striated muscle cell differentiation further implies a role in muscle development. However, reliance on GO annotations derived from model organisms may overlook parasite-specific phosphorylation mechanisms. Future studies could explore the functional role of these enriched pathways in parasite-specific contexts.

### Enriched pathways of *B. procyonis* phosphoproteins

**Fig. 3:**
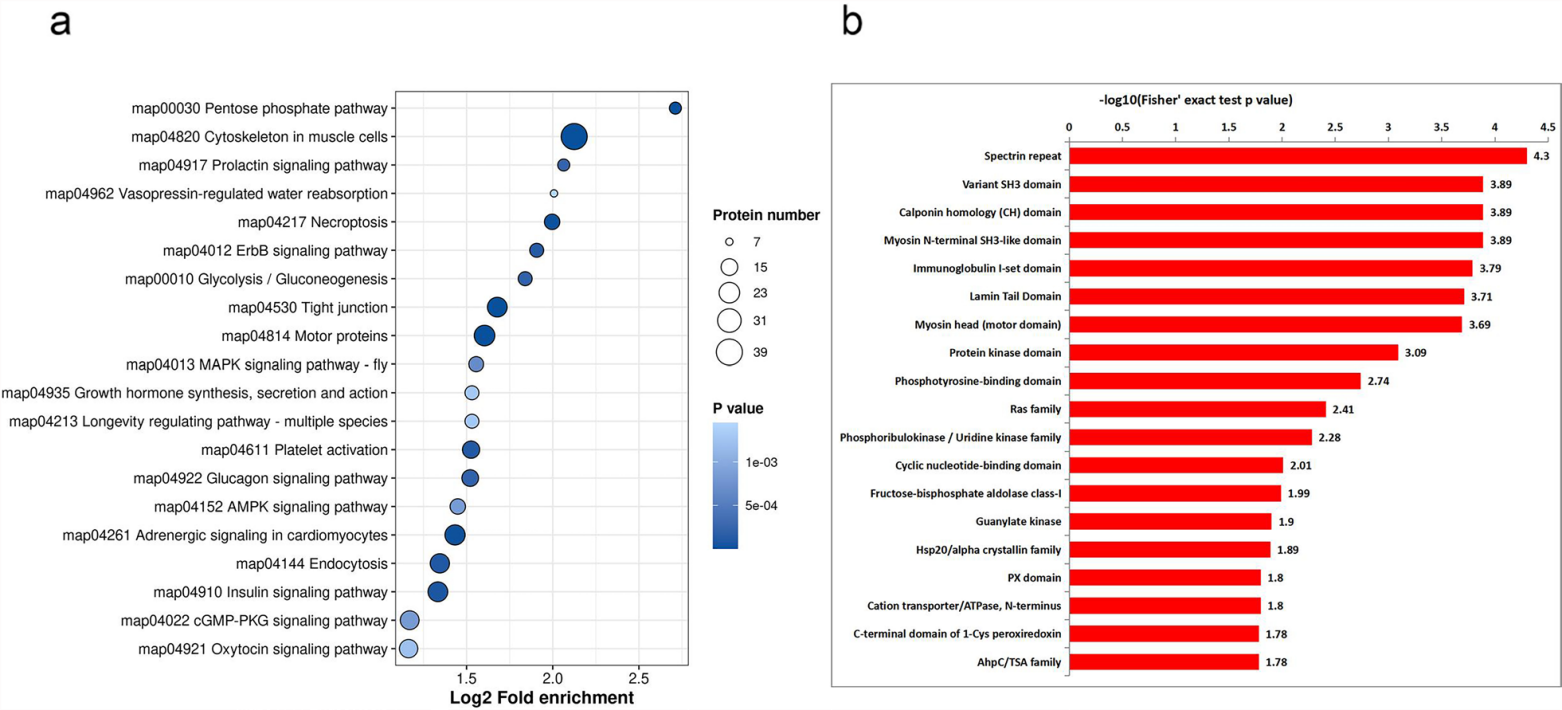
Enrichment analysis of KEGG (**a**) and domain analysis (**b**) of *Baylisascaris procyonis* phosphoproteome

According to the analysis against the KEGG database, among the top 20 significantly enriched pathways in *B. procyonis*, the insulin signaling pathway was particularly important (Fig. 3a and Additional file 10: Table S7). This signaling pathway is an integral, evolutionary conserved pathway for metazoan development, metabolism, and behavior [53]. The insulin signaling pathway starts with the binding of insulin to the insulin receptor (IR), a tyrosine kinase on the cell surface. The insulin receptor kinase (IRK) is then activated and autophosphorylated to tyrosine phosphorylate the cellular substrates required for insulin response entrainment [54]. Pierce et al. [55] identified 37 genes predicted to encode insulin-like peptides in the model organism *C. elegans*, which suggested the diversification and importance of the insulin superfamily. The high enrichment of phosphorylated proteins within this pathway strongly implicates post-translational modification as a core regulatory mechanism for metabolic adaptation and development in *B. procyonis*.

Additionally, phosphoproteomic analysis revealed enrichment of pathways including tight junction (TJ) regulation, endocytosis, and longevity signaling in *B. procyonis*. Among these, endocytosis emerged as a critical process, enabling eukaryotic parasites to maintain membrane homeostasis, recycle receptors, and acquire exogenous nutrients [56]. For instance, *Plasmodium* spp. internalize host hemoglobin via endocytic vesicles to support amino acid metabolism, while *Toxoplasma gondii* actively engulfs cytoplasmic components through an ingestion pathway [57]. Notably, endocytosis is also implicated in extracellular vesicle (EV) uptake. EVs can regulate macrophage activation and inflammatory responses and contribute to host-parasite interactions and intracellular communication [58]. A variety of studies on parasites, including *Ascaris suum*, *Oesophagostomum dentatum*, and *Trichuris suis*, have proved the EV uptake by host cells. The enrichment of endocytosis in *B. procyonis* suggests potential EV-mediated communication or nutrient scavenging during infection. The significant enrichment of the tight junction (TJ) pathway highlights parasite-host interface interactions. Phosphorylation likely plays a key role in dynamically regulating TJ complex assembly and permeability within the parasite’s epidermal or intestinal barriers. TJ complexes maintain intestinal barrier integrity through regulated endocytosis and lysosomal trafficking [59]. Similarly, *Trichinella spiralis* secretes proteases that degrade TJ components, such as E-cadherin, and alters claudin expression, promoting larval migration [60]. The co-enrichment of TJ and endocytosis pathways in *B. procyonis* implies a coordinated strategy for breaching host barriers, potentially involving protease-mediated TJ remodeling and vesicle trafficking. The prominence of both insulin signaling and TJ regulation in the phosphoproteome underscores their critical, phosphorylation-dependent roles in *B. procyonis* biology. These findings position endocytosis and TJ modulation as central mechanisms for *B. procyonis* to interact with host environments, suggesting conserved yet adaptable strategies across parasitic nematodes.

We used the InterPro program to predict important domains and categorize the phosphoproteins into families (Fig. 3b, Additional file 11: Table S8). Among the different domains found in phosphoproteins, several were significantly enriched, including the spectrin repeat, variant SH3 domain, calponin homology (CH) domain, myosin N-terminal SH3-like domain, and immunoglobulin I-set domain. Particularly notable was the significant enrichment of the Src homology 3 (SH3) domain and its variant. SH3 domains were first identified as a predominant protein module distinguishing proline-rich sequences, especially those with a PxxP motif. These domains participate in several essential cellular processes, such as intracellular signaling and cellular environment communication, cell development and differentiation, cytoskeletal reorganization and cell motility, immune responses, and protein trafficking and degradation [61–64]. The phosphorylation of this domain could be essential for recognizing ssRNA or ssDNA and regulating multiple protein domain interactions. These enriched domains collectively highlight core adaptive mechanisms in *B. procyonis* related to dynamic cytoskeletal regulation (spectrin/CH domains) and stress responses, providing new perspectives for understanding its parasitic biology.

### Protein-protein interaction network

**Fig. 4:**
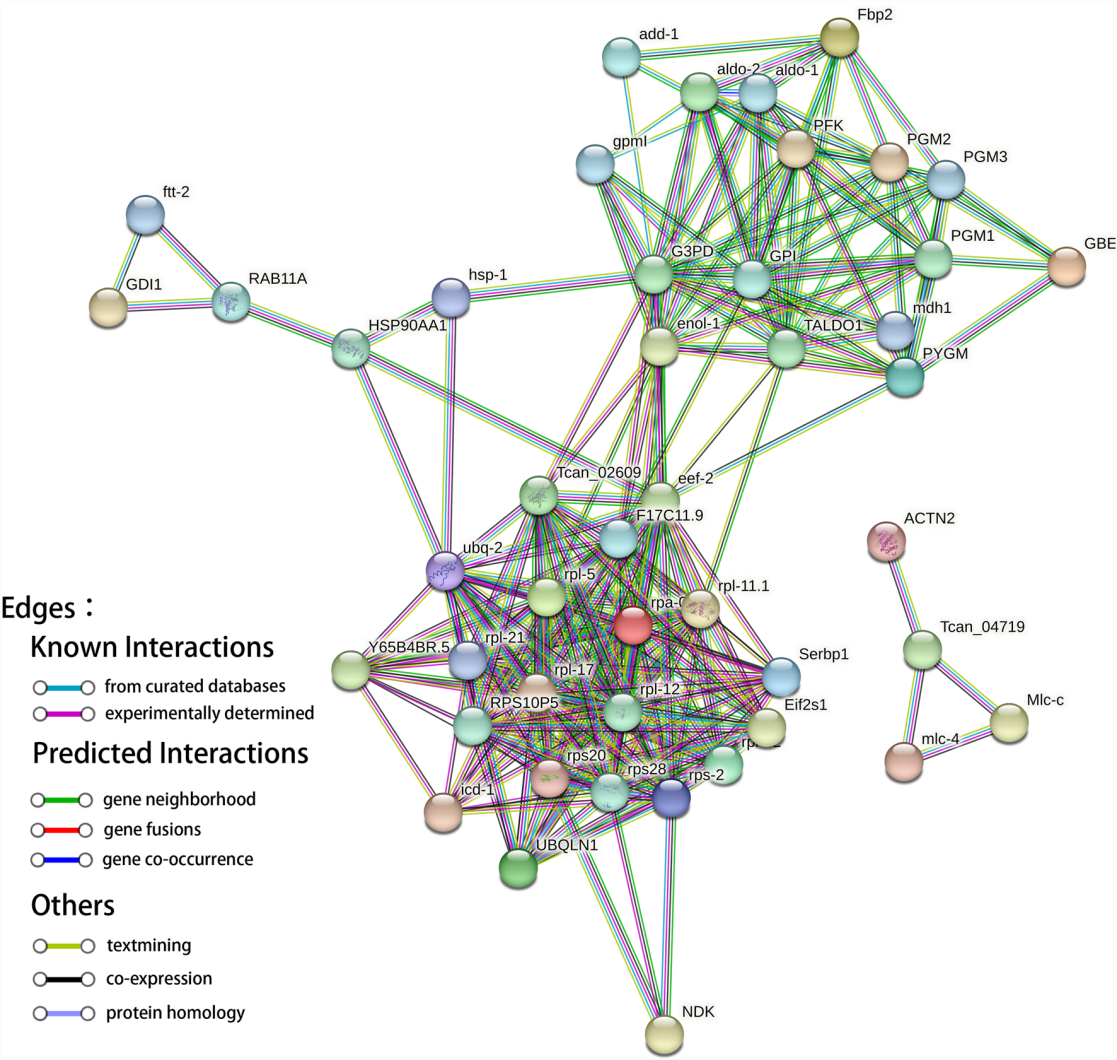
Phosphoprotein networks resulting from the *Baylisascaris procyonis* gene network

**Fig. 5:**
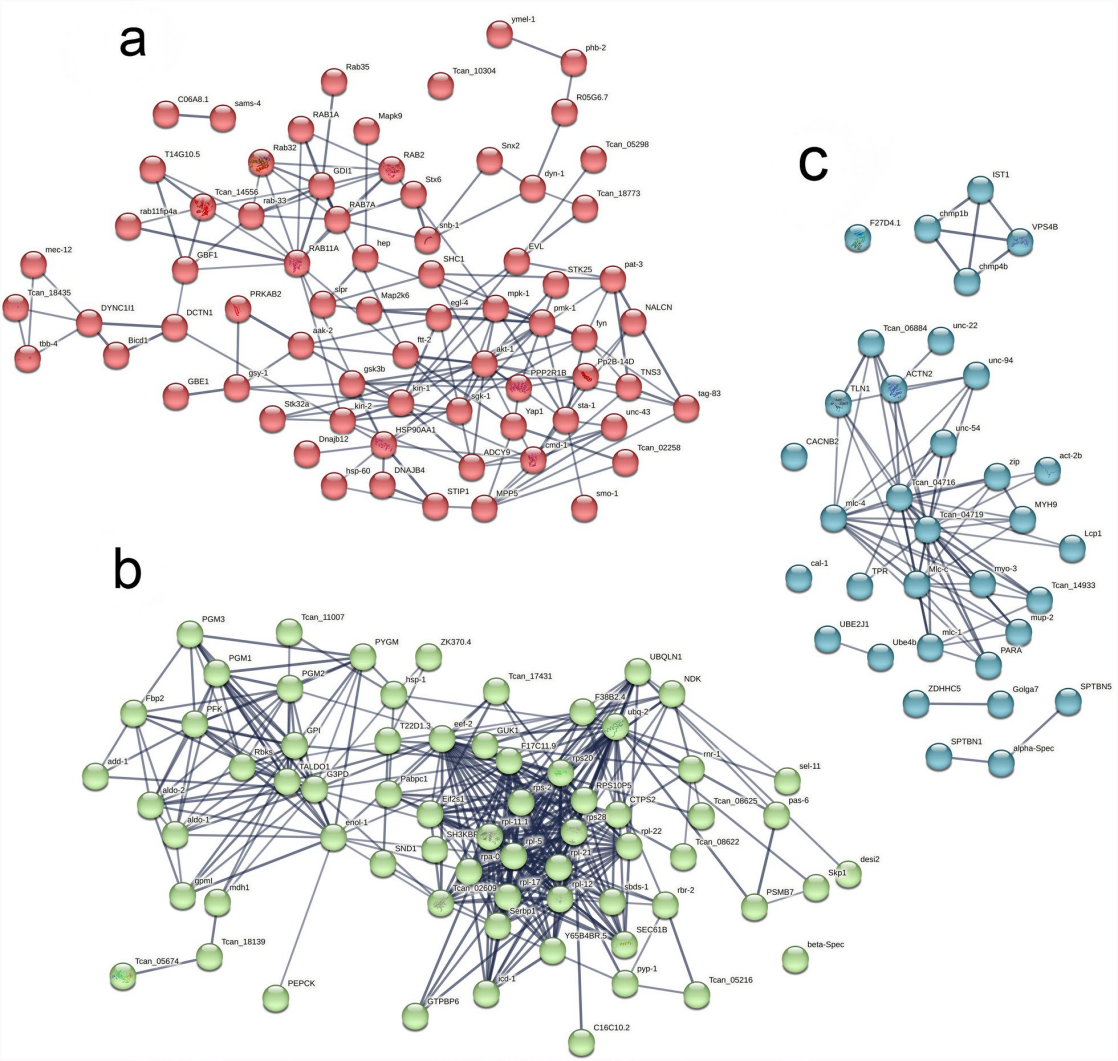
*Baylisascaris procyonis* phosphoprotein interaction network. (**a**) Sub-clusters of PPI network; Cluster 1, nodes: 75; edges: 480; *P*-value: < 1.0e-16. (**b**) Sub-clusters of PPI network; Cluster 2, nodes: 69; edges: 607; *P*-value: < 1.0e-16. (**c**) Sub-clusters of PPI network; Cluster 3, nodes: 43; edges: 192; *P*-value: < 1.0e-16

Figure 4 presents a protein-protein interaction network constructed after filtering and mapping the top 50 significant interactions. The final phosphoprotein interactome held 50 nodes and 94 interactions (edges), presenting a highly dense connection feature with an average node degree of 10.6. The core hub nodes, such as ubq-2 (with the highest connectivity) and ribosomal protein complex members (including rpl-11.1, rpl-5, and rps20), are significantly enriched in the network. The surrounding nodes, particularly the translation elongation eef-2, form tight modules through thick edges (high-confidence interactions), suggesting the enrichment of the core functions of ubiquitination modification and ribosome assembly. Node clustering analysis shows that the interaction network can be divided into three functional modules (Fig. 5). Functional annotation showed that Cluster 1 (Fig. 5a) included 75 enriched proteins (out of 187 total) involved in the proteasome, protein processing in the endoplasmic reticulum (ER), and ErbB signaling pathways, with atk-1 as the core protein. Cluster 2 (Fig. 5b) aggregated 69 enriched proteins (out of 187 total) associated with the ribosome, protein export, and ribosome biogenesis pathways, featuring ubq-2 as the core protein. Cluster 3 (Fig. 5c) enriched 43 proteins (out of 187 total) contributing to fructose and mannose metabolism, pentose phosphate pathway, arginine biosynthesis, and glycolysis/gluconeogenesis, with Tcan_04719 as the core protein. Notably, the core protein ubq-2 represents a promising candidate drug target due to its central regulatory role in protein quality control and degradation. Furthermore, the functional and pathway enrichment profiles of these core proteins (including ubq-2,atk-1, and Tcan_04719) exhibit significant convergence with key metabolic pathways such as insulin signaling and glycolysis, as previously identified in functional enrichment analyses of phosphoproteins. This provides critical insights into signaling and metabolic mechanisms of *B. procyonis*, facilitating a deeper understanding of its infection strategies and survival tactics.

## Conclusions

In this study, a qualitative phosphoproteomics approach was employed to analyze the protein phosphorylation modification network in *B. procyonis*. This research identified 3308 phosphopeptides with 854 phosphorylation sites across 450 proteins, spanning diverse functional categories critical for parasite motility and structural regulation. Functional annotation revealed 77.52% phosphoserine dominance and significant enrichment in cytoskeletal dynamics as well as host-parasite interaction processes. The identified phosphorylated proteins and associated signaling pathways may serve as potential therapeutic targets. Subsequent studies should focus on validating their regulatory mechanisms and biological functions in parasite survival and transmission.

## Supplementary Information

Additional file 1. Fig. S1 Subcellular location of phosphorylated proteins.

Additional file 2. Text S1. Rapid molecular verification. Fig. S2. Specific 200-bp amplicon of *Baylisascaris procyonis* *rrnL*.

Additional file 3. Dataset S1. The protein library for identifying phosphopeptides.

Additional file 4. Table S1. Overview of protein identification.

Additional file 5. Table S2. Summary of MS/MS spectrum database search analysis.

Additional file 6. Table S3. GO terms for the *Baylisascaris* *procyonis* phosphoproteins.

Additional file 7. Table S4. Subcellular localization prediction of *Baylisascaris* *procyonis* phosphoproteins.

Additional file 8. Table S5. The motif analysis of *Baylisascaris* *procyonis*.

Additional file 9. Table S6. The GO analysis of *Baylisascaris* *procyonis* phosphoproteins.

Additional file 10. Table S7. The KEGG pathway enrichment of *Baylisascaris* *procyonis* phosphoproteins.

Additional file 11. Table S8. The protein domain enrichment of *Baylisascaris* *procyonis* phosphoproteins.

## Data Availability

Data supporting the main conclusions of this study are included in the manuscript or supplementary information files.

## Abbreviations

BCA: Bicinchoninic acid
IMAC: Immobilized metal affinity chromatography
LC-MSC-MS/MS: Liquid chromatography-tandem mass spectrometry
MS: Mass spectrometry
UPLC: Ultra-performance liquid chromatography
HCD: Higher-energy C-trap dissociation
NCE: Nominal collision energy
AGC: Automatic gain control
PSM: Peptide-spectrum match
GO: Gene Ontology
KEGG: Kyoto Encyclopedia of Genes and Genomes
