# Supplementary figures and images for: Protein phosphorylation networks in Baylisascaris procyonis revealed by phosphoproteomic analysis
Source: Parasit Vectors. 2025 Jul 28;18:307. doi: 10.1186/s13071-025-06949-y (PMC12305901; doi:10.1186/s13071-025-06949-y)

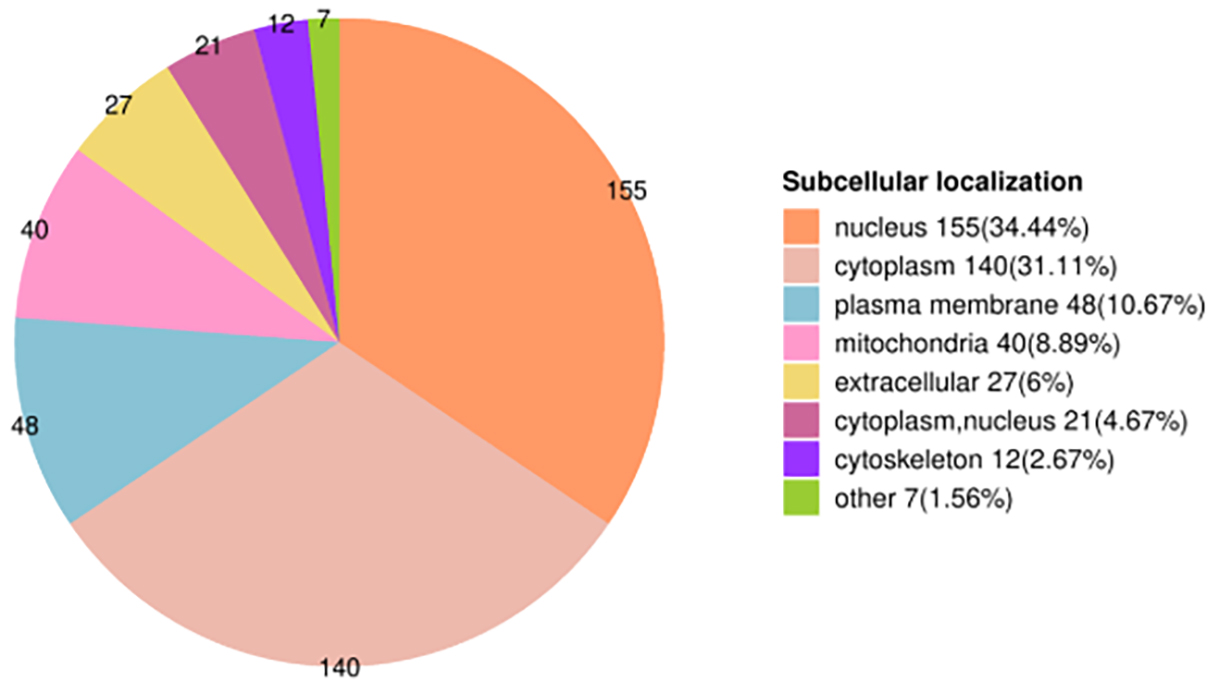

Supplement: Supplementary file 3 — Additional file 3. Dataset S1. The protein library for identifying phosphopeptides. [file 13071_2025_6949_MOESM3_ESM.jpg]

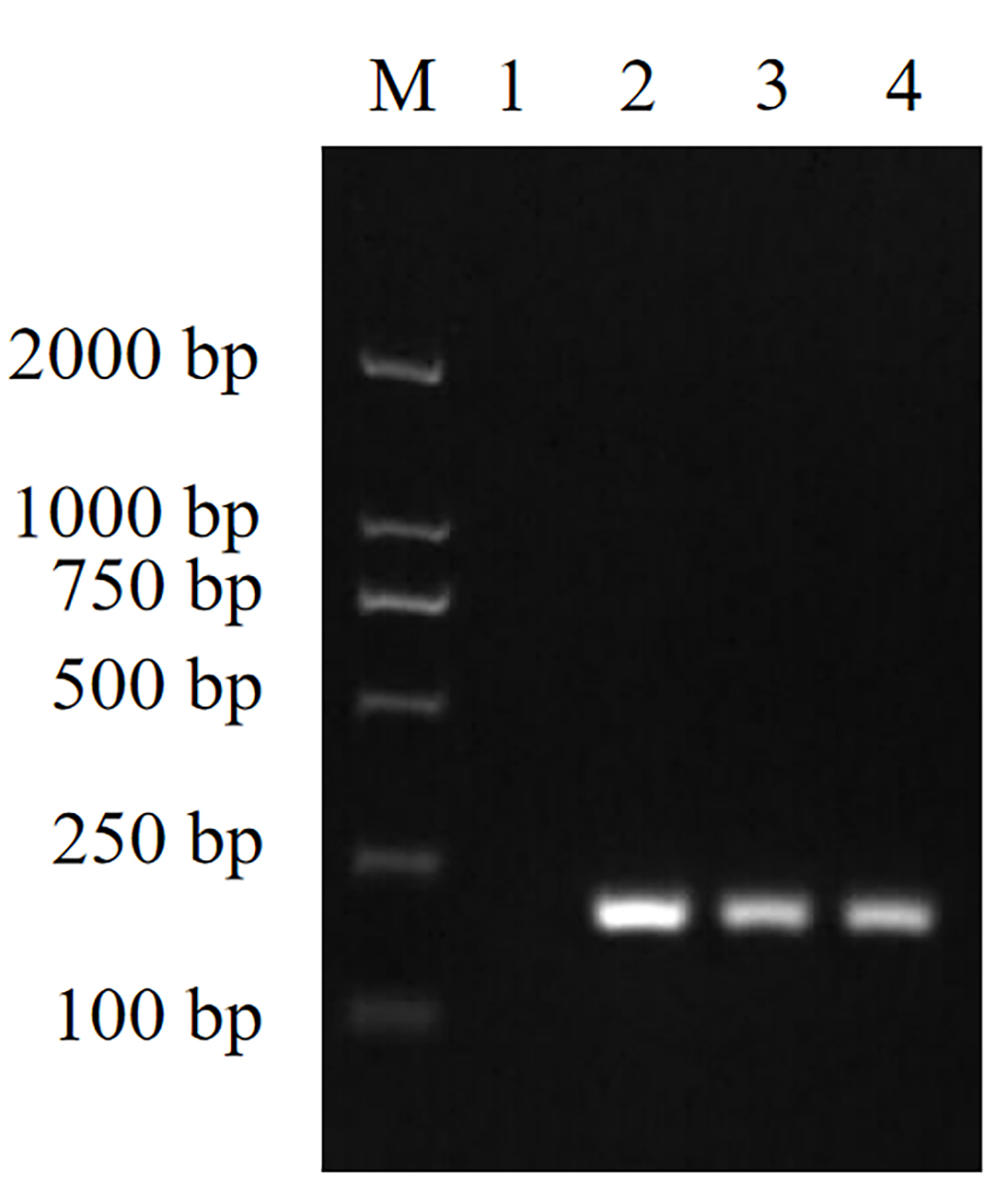

Supplement: Supplementary file 4 — Additional file 4. Table S1. Overview of protein identification. [file 13071_2025_6949_MOESM4_ESM.zip › New folder/Fig. S2.jpg]
