# Supplementary material for: Protein phosphorylation networks in Baylisascaris procyonis revealed by phosphoproteomic analysis
Source: Parasit Vectors. 2025 Jul 28;18:307. doi: 10.1186/s13071-025-06949-y (PMC12305901; doi:10.1186/s13071-025-06949-y)
Supplement: Supplementary file 4 — Additional file 4. Table S1. Overview of protein identification. [file 13071_2025_6949_MOESM4_ESM.zip › New folder/Text S1.doc]

**Materials and Methods**

**Sample Isolation**

The three *Baylisascaris procyonis* samples in this study were collected from the intestine of a raccoon from Changsha Ecological Zoo in Hunan Province. After washing, the worms were stored in 70% alcohol at -20℃.

**DNA Extraction and Enzymatic Amplification**

Genomic DNA was extracted from adult *B. procyonis* samples using the DNeasy Blood & Tissue Kit (Qiagen, Beijing, China). Species identification was performed via PCR amplification of the mitochondrial *rrnL* (200 bp) gene (primers[1]: *rrnL*-F: 5’-GAGAACTGGCGGGG-3’, *rrnL*-R: 5’-CTCACACTGACTTACACACC-3’). The PCR amplification system was 25 µL: 12.5 µL Taq PCR Master Mix (Bao Bioengineering Co., Dalian, China), 9.5 µL ddH2O, upstream and downstream primers (100 pmol /µL, 0.5 µL each), and 2 µL template DNA. At the same time, double distilled water was used as blank control instead of template DNA. PCR amplification conditions were as follows: pre denaturation at 94℃ for 4 min; denaturation at 94℃ for 30 s, annealing at 55℃ for 30 s, and extension at 72℃ for 45 s, 35 cycles; and final extension at 72℃ for 10 min. Each PCR yielded a single band, detected in a 1% (W/V) agarose gel stained with ethidium bromide.

**Results**

As shown in **Fig. S2**, PCR verification of all three biological replicates yielded specific 200-bp amplicons corresponding to *B. procyonis* mitochondrial *rrnL*, while negative controls remained blank. Our rapid molecular verification unequivocally identifies the specimen as *B. procyonis* (raccoon roundworm).

**References**

1.	Xie Y, Zhang Z, Niu L, Wang Q, Wang C, Lan J, et al. The mitochondrial genome o*f Baylisascaris procyon*is. PLoS One. 2011;6(10):e27066.

**Figure legends:**

**Fig. S2** Specific 200-bp amplicon of *B. procyonis rrnL* M: DL2000 molecular marker; 1: negative control; 2-4: *B. procyonis* samples
